# Protein coding variation in the J:ARC and J:DO outbred laboratory mouse stocks provides a molecular basis for distinct research applications

**DOI:** 10.1093/g3journal/jkad015

**Published:** 2023-01-17

**Authors:** Belinda K Cornes, Carolyn Paisie, Emily Swanzey, Peter D Fields, Andrew Schile, Kelly Brackett, Laura G Reinholdt, Anuj Srivastava

**Affiliations:** Mammalian Genetics, The Jackson Laboratory, 600 Main Street, USA; Mammalian Genetics, The Jackson Laboratory, 600 Main Street, USA; Mammalian Genetics, The Jackson Laboratory, 600 Main Street, USA; Mammalian Genetics, The Jackson Laboratory, 600 Main Street, USA; Mammalian Genetics, The Jackson Laboratory, 600 Main Street, USA; Mammalian Genetics, The Jackson Laboratory, 600 Main Street, USA; Mammalian Genetics, The Jackson Laboratory, 600 Main Street, USA; Mammalian Genetics, The Jackson Laboratory, 600 Main Street, USA

**Keywords:** outbred stocks, laboratory mice, reproducibility, exome, genetic quality control

## Abstract

Outbred laboratory mice (*Mus musculus*) are readily available and have high fecundity, making them a popular choice in biomedical research, especially toxicological and pharmacological applications. Direct high throughput genome sequencing (HTS) of these widely used research animals is an important genetic quality control measure that enhances research reproducibility. HTS data have been used to confirm the common origin of outbred stocks and to molecularly define distinct outbred populations. But these data have also revealed unexpected population structure and homozygosity in some populations; genetic features that emerge when outbred stocks are not properly maintained. We used exome sequencing to discover and interrogate protein-coding variation in a newly established population of Swiss-derived outbred stock (J:ARC) that is closely related to other, commonly used CD-1 outbred populations. We used these data to describe the genetic architecture of the J:ARC population including heterozygosity, minor allele frequency, LD decay, and we defined novel, protein-coding sequence variation. These data reveal the expected genetic architecture for a properly maintained outbred stock and provide a basis for the on-going genetic quality control. We also compared these data to protein-coding variation found in a multiparent outbred stock, the Diversity Outbred (J:DO). We found that the more recently derived, multiparent outbred stock has significantly higher interindividual variability, greater overall genetic variation, higher heterozygosity, and fewer novel variants than the Swiss-derived J:ARC stock. However, among the novel variants found in the J:DO stock, significantly more are predicted to be protein-damaging. The fact that individuals from this population can tolerate a higher load of potentially damaging variants highlights the buffering effects of allelic diversity and the differing selective pressures in these stocks. While both outbred stocks offer significant individual heterozygosity, our data provide a molecular basis for their intended applications, where the J:DO are best suited for studies requiring maximum, population-level genetic diversity and power for mapping, while the J:ARC are best suited as a general-purpose outbred stock with robust fecundity, relatively low allelic diversity, and less potential for extreme phenotypic variability.

## Introduction

There are 1,374 inbred strains and outbred laboratory mouse stocks cited in the biomedical research literature (BULT *et al.* 2019). Genome sequencing of inbred strains has confirmed the expected homozygosity and intrastrain genetic divergence that are the results of decades of inbreeding and reproductive isolation (Mouse Genome Sequencing *et al.* 2002; Keane *et al.* 2011; Yalcin *et al.* 2011; Chang *et al.* 2017; Srivastava *et al.* 2017; Lilue *et al.* 2018). Because inbred mice are genetically identical, interindividual phenotypic variability can be attributed to experimental variables and to a lesser extent, epigenetic variation (Weichman and Chaillet 1997). These unique features make inbred mouse strains a popular choice for biomedical researchers who endeavor to minimize genetic variability and quantify experimental (extrinsic, non-genetic) variability. However, inbred strains do not recapitulate the extent of interindividual variation found in a human cohort, patient group, or population making them inappropriate for studies that seek to model such variation.

In contrast to inbred strains, outbred stocks exhibit interindividual variation and heterozygosity, coupled with higher fecundity. The four most widely cited outbred stocks are CF-1, Swiss Webster, NMRI, and CD-1 (Bult *et al.* 2019), but all trace their origins to a colony at The Rockefeller Institute that was established with 2 male and 7 female “Swiss’ mice imported from the Pasteur Institute in 1926 (Chia *et al.* 2005). Over the ensuing decades, mice from these stocks were imported by commercial breeders, NIH, and academic institutions, where colonies were ideally maintained with at least 25 breeding pairs to maintain a coefficient of inbreeding (F) of less than 1% per generation with selection for maximum fecundity (Festing *et al.* 1972) (Chia *et al.* 2005). A variety of approaches have been used to examine the genetic variation resident in these outbred stocks including alloenzyme analysis and other biochemical approaches (Groen and Lagerwerf 1979; Rice and O'Brien 1980; Cui *et al.* 1993), high-density SNP panels (Aldinger *et al.* 2009; Yalcin *et al.* 2010), and most recently, low coverage genome sequencing (Yalcin *et al.* 2010; Nicod *et al.* 2016). Data from these studies provide estimates of minor allele frequency (MAF), heterozygosity, and coefficients of inbreeding. Specifically, low coverage sequencing of these stocks has revealed 1) relatively low allelic diversity given their common origin, 2) appreciable levels of heterozygosity of common variants (0.20–0.35) in properly maintained populations, and 3) relatively few novel variants (<5%) relative to the C57BL/6J reference genome and other common inbred laboratory strains (Yalcin *et al.* 2010; Nicod *et al.* 2016).

The JAX Swiss Outbred stock was imported to The Jackson Laboratory (JAX) from The Animal Resources Centre (ARC) in Canning Vale, Western Australia in 2020 as a group of 128 individuals (64 male and 64 female). To fully characterize the extent of protein-coding variation in this new outbred population, we sequenced the exomes of 32 male and 32 female G3 animals that were used to initiate new breeding funnels at JAX (J:ARC). We used these data to estimate inbreeding, heterozygosity, and interindividual variation, and to molecularly define the colony. These metrics confirmed the outbred nature of this new colony of CD-1 stock, and additionally reveal 5,105 (3.8%) novel protein-coding and splice site variants, which falls in the range of the proportion of novel variants found in other outbred colonies and stocks (Yalcin *et al.* 2010; Nicod *et al.* 2016). To provide a molecular genetic quality control standard for outbred stocks, we compared the J:ARC exome call sets to exome variant call sets from a very different outbred stock, derived from a multiparent outbred population, the Diversity Outbred (J:DO). We found that the J:DO have higher population level genetic variation (∼3.6X), higher interindividual variation, higher heterozygosity, and higher allelic diversity due to their multi-substrain parental origins. We found fewer novel variants in the J:DO, yet these novel variants are predicted to have a higher impact on protein function. These common and distinct genomic features of the J:ARC and J:DO can serve as the basis for estimating sample size, where fewer J:ARC mice are needed to capture the full range of segregating variation in the population, but a similarly sized cohort of J:DO mice offers more interindividual genetic variation and a higher population level genetic variation. Both types of outbred stocks can be used for genetic mapping; however, their distinct genetic architectures must be accounted for in the study design as previously shown (Gatti *et al.* 2014; Nicod *et al.* 2016), and should be regularly monitored to ensure reproducibility.

## Materials and methods

### Samples

**Fig. 1.**
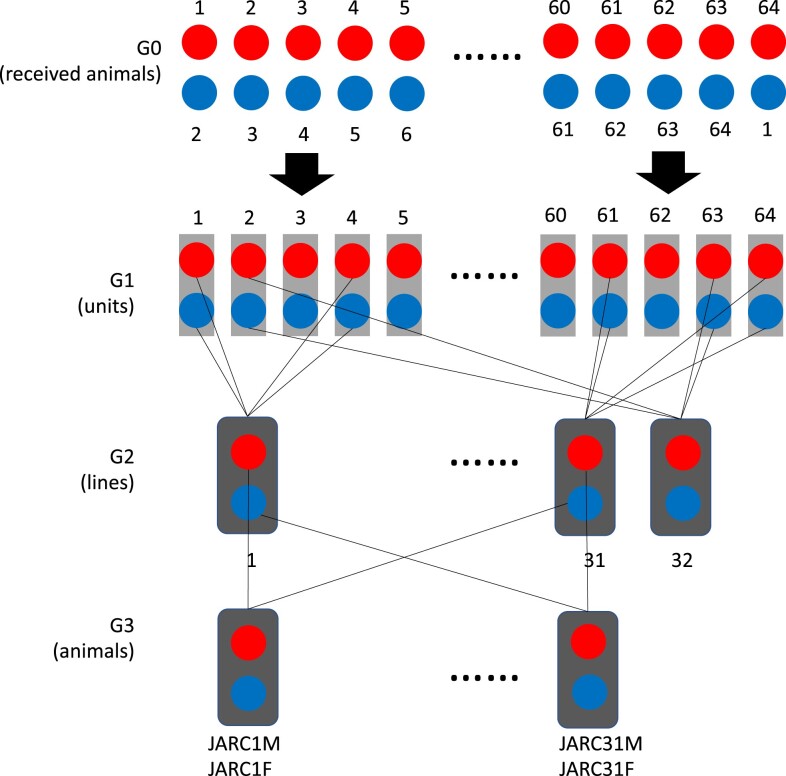
The Poiley method used for the importation and breeding of the JAX Swiss Outbred (J:ARC) population. G0 are live animals from The Animal Resources Centre (ARC) in Canning Vale that are subsequently bred and rederived through IVF to create 32 distinct breeding lines at The Jackson Laboratory which continue to be maintained according to the Poiley method to minimize inbreeding.

*J:ARC(S), JAX Swiss Outbred.* The JAX Swiss Outbred stock was imported to The Jackson Laboratory from The ARC, Canning Vale, Western Australia in 2020. The imported mice (G0) were paired into 64 breeding units, and sperm and eggs were harvested from the G1 offspring. To establish each of the 32 breeding funnels for live colony maintenance in the JAX vivarium barrier, two distinct units were selected for reciprocal in vitro fertilization and IVF-generated embryos were pooled. The resulting live-born animals from each funnel were designated G2. These mice are subsequently bred according to the Poiley rotational breeding scheme to produce 32 breeding funnels at G3 (Poiley 1960) (Fig. 1). Spleen samples are collected from one male and one female sibling representing each of these breeding funnels for a total of 64 G3 samples J:ARC (J:ARC(S), RRID:IMSR_JAX:034608, JAX stock 034608) (32 females and 32 males) (Supplementary File 1).

*J:DO, JAX Diversity Outbred.* We collected spleen from 20 randomly selected J:DO, Diversity Outbred (RRID:IMSR_JAX:009376, JAX stock 009376) mice from The Jackson Laboratory, 10 female and 10 male, nonsibling mice, breeding generation 43 (Supplementary File 1).

### DNA isolation, exome library preparation, and sequencing

DNA isolation, exome library preparation, and sequencing were performed by Genome Technologies at The Jackson Laboratory. DNA was isolated from the spleen using the NucleoMag Tissue Kit (Machery-Nagel) according to the manufacturer’s protocol. DNA concentration and quality were assessed using the Nanodrop 8000 spectrophotometer (Thermo Scientific), the Qubit Flex dsDNA BR Assay (Thermo Scientific), and the Genomic DNA ScreenTape Analysis Assay (Agilent Technologies). Mouse exome libraries were constructed using the KAPA HyperPrep Kit (Roche Sequencing and Life Science) and SureSelect XT Community Design Mouse All Exon v2 Target Enrichment System (Agilent Technologies), according to the manufactures’ protocols. Briefly, the protocol entails shearing the DNA using the E220 Focused-ultrasonicator (Covaris), size selection targeting 400 bp, ligating Illumina-specific adapters, and PCR amplification. The amplified DNA libraries are then hybridized to the Mouse All Exon probes (Agilent Technologies) and amplified using indexed primers. The quality and concentration of the libraries were assessed using a High Sensitivity D1000 ScreenTape (Agilent Technologies) and KAPA Library Quantification Kit (Roche Sequencing and Life Science), respectively, according to the manufacturers’ instructions. Libraries were sequenced 150 bp paired-end on an Illumina NovaSeq 6000 using the S4 Reagent Kit v1.5.

### Sequence data analysis

An overview of the sequence data analysis pipeline is shown in Supplementary Fig. 1 and the pipeline itself is available at https://github.com/TheJacksonLaboratory/cs-nf-pipelines (−workflow wes and –gen_org mouse). All reads were subjected to quality control using an in-house QC script. Samples with base qualities greater ≥30 over 70% of read length were used in the downstream analysis. High-quality reads were mapped to the mouse genome (build-mm10) using BWA-mem (bwa-0.7.9a) at default parameters (Aldinger *et al.* 2009). The resulting alignment was sorted by coordinates and converted to binary alignment map (BAM) format by Picard v 2.8.1- SortSam utility (http://picard.sourceforge.net). The Picard-MarkDuplicates module was used to remove duplicates from the data. The Genome Analysis Toolkit (GATK v4) (McKenna *et al.* 2010; DePristo *et al.* 2011) module BaseRecalibrator was used to preprocess the alignments. Target capture efficiency was determined using Picard-HsMetrics (1.95). The recalibrated bam alignment file was used to input GATK-Haplotype Caller at parameters -stand_call_conf 50.0, -stand_emit_conf 30.0, and variants calls were restricted to the target region (Mouse All Exon v2). Finally, raw variants calls were soft filtered using GATK VariantFiltration (DP < 25, QD < 1.5 and FS > 60), annotated by snpEff 3.6.c (Cingolani *et al.* 2012) and the highest impact variant was reported by GATK VariantAnnotator. All variants were further annotated with mouse dbSNP v150.

GATK-HaplotypeCaller in the GVCF mode was used for joint genotyping of J:DO, J:ARC, and combined (J:DO and J:ARC) samples. The CombineGVCF utility was used to gather all the samples and then to execute the GenotypeGVCF command. All analyses were performed on jointly called variants and only included variants found in both “J:ARC only” and “J:DO only” called datasets (N:120,862). This effectively revealed the differences between the two datasets and directly provided the data needed to resolve genotypes.

### Heterozygosity

To assess the distribution of mean heterozygosity across all samples, Plink was used (Purcell *et al.* 2007). The “–het” option in Plink was used to create heterozygous information for each sample which included the observed number of homozygous genotypes ”[O(Hom)]” and the number of nonmissing genotypes “[N(NM)]”. This information was then used to calculate the observed heterozygosity rate per individual using the formula “(N(NM) - O(Hom))/N(NM)”.

To calculate per variant heterozygosity for variants shared between J:DO and J:ARC, the number of samples in which a shared variant was heterozygous was divided by the total number of genotyped samples. The inbreeding coefficient (FH), was calculated using VCFtools (−het) as previously described (Stoffel et al. 2016; Foster et al. 2021). Plink was used to calculate r2 for all pairs of autosomal SNPs called from joint genotyping in the J:ARC samples (425,409) and the J:DO samples (117,429). SNPs that were missing in more than 5% of the samples and that were monomorphic were removed. The 95th percentile of r2 values for SNPs spaced up to 1Mb apart was selected.

### Allele frequency

To observe differences in allele frequencies across the genome between J:DO and J:ARC samples, the MAF was calculated separately for the J:DO and J:ARC samples at each variant site using the “–freq” option in Plink. To compare differences in allele frequencies between males and females in each population, we calculated the XtX statistic, a locus-specific F_ST_ corrected for the covariance of population/grouping allele frequencies, first introduced by Gunther and Coop (2013) and estimated with BayPass (Gautier 2015). Specifically, we used the “core” model of BayPass with default parameters (i.e. MCMC settings of 20 pilot runs of 500 iterations, a burn-in of 5,000 iterations, and a retained sample of 1,000 iterations with a thinning interval of 20; three independent runs were made to assess convergence of results) to estimate the XtX for all variants that were biallelic, genotyped in all individuals, and occurred on autosomes. A histogram of *P*-values for the XtX summary was assessed prior to the false-discovery rate (FDR) control. To control for the large number of tests contained in our XtX analysis we applied Benjamini–Hochberg (BH) (Benjamini and Hochberg 1995) correction to our BayPass derived *P*-values using the R package qvalue v.2.30.0 (http://github.com/jdstorey/qvalue) with a FDR threshold of 0.05.

### Clustering/dendrogram

Principal component analysis (PCA) plots were created in Plink. PCA is a multivariate statistical method used to produce any number of uncorrelated variables (or principal components) from a data matrix containing observations across a number of potentially correlated variables. The principal components were calculated so that the first principal component accounts for as much variation in the data as possible in a single axis of variation (component), followed by additional components. Here, the variants called in both the J:ARC and J:DO datasets were used to observe the similarities within and between datasets.

To show similarity groups among the J:ARC samples, a dendrogram was created using the ggdendro package in R-3.6.2 (https://cran.r-project.org). A dendrogram is a tree diagram showing hierarchical clustering that represents the relationships of similarity among a group of entities.

### Novel variants

The joint genotyping variant call file (vcf) of the J:ARC and J:DO were flagged for known variants in dbSNP150 (Sherry *et al.* 2001), European Variation Archive (EVA) (Cezard *et al.* 2022), Sanger mouses genome project (Keane *et al.* 2011) (ftp://ftp-mouse.sanger.ac.uk//REL-2004-v7-SNPs_Indels/mgp_REL2005_snps_indels.vcf.gz), mouse collaborative cross (CC) genome (ftp://ftp.sra.ebi.ac.uk/vol1/ERZ460/ERZ460702/Merged_69_flagged.tab.vcf.gz) (Srivastava *et al.* 2017), and in-house variant calls generated from Diversity Outbred low-pass sequencing (Morgan *et al.* 2017) using vcftools “vcf-annotate” (Keane *et al.* 2011) and snpsift_4.2 (Cingolani *et al.* 2012). We also flagged the J:ARC variants for their presence in the J:DO joint and single sample variant call sets and did a similar analysis for the J:DO variants. The variants not present in any of these resources were considered novel and further annotated by snpeff v4.3 (Cingolani *et al.* 2012) using the mouse GRCm38.75 snpeff database. Finally, the highest effect variants are selected by gatk-3.6 VariantAnnotator (McKenna *et al.* 2010). Functional gene and pathway annotations (including KEGG, GO, UP, BIOCARTA, REACTOME) for the genes harboring novel high-impact variants were compiled using DAVID Bioinformatics tools. DAVID Bioinformatics tools were then used to identify clusters of genes with related functional annotations using a medium classification stringency and a Benjamini score of >0.05 as the significance threshold for enrichment (Sherman *et al.* 2022).

## Results and discussion

The J:ARC outbred stock currently maintained at The Jackson Laboratory was established through the importation of mice from the Australian Animal Resource Center (ARC) in 2020. The origins of this stock are CD-1 mice that were acquired by ARC from Charles River Laboratories in 1991 and 2005. Like most outbred laboratory mice, CD-1 mice trace their origins to 2 male and 7 female “Swiss” mice that were obtained by The Rockefeller Institute in 1926 (Chia *et al.* 2005). The live J:ARC colony at the Jackson Laboratory was initiated with 64 founder animals (G3) in 32 breeding funnels. To characterize allelic diversity in this new outbred colony and to create a reference set of variants for future genetic monitoring (Strobel *et al.* 2015), we sought to catalog the founder alleles with a focus on protein-coding variants. To do this, we harvest tissue from the G3 siblings that are randomly bred to create each breeding funnel [1 male (JARC1M) and 1 female (JARC1F) per funnel] (Fig. 1). We also wanted to compare the genetic architecture of the outbred J:ARC to a multiparent outbred population (J:DO) to demonstrate the differing genetic architectures of these stocks. J:DO mice are derived from eight founder inbred strains representing the three major subspecies of laboratory mice, *M.m.musculus, M.m.domestics*, and *M.m.castaneous*. This population captures 90% of the genetic variation in laboratory mice and with each generation of breeding, sufficient accumulated recombination to ensure high LD decay, and thereby, maximum power for genetic mapping (Gatti *et al.* 2014; Saul *et al.* 2019). The J:DO is maintained at the Jackson Laboratory as 175 breeding funnels using MateSel (Kinghorn and Kinghorn 2021) to minimize inadvertent phenotypic selection, optimize diversity, and prevent the intracolony structure in each generation based on pedigreed population history. Whole genome variant (WGS) call sets from the recombinant inbred founders (CC, the collaborative cross) of the J:DO and from an earlier generation of the J:DO (G < 30) have been described (Morgan *et al.* 2017; Srivastava *et al.* 2017). To augment these call sets and to provide a snapshot of interindividual variation at a more advanced breeding generation (G43), we sampled 20 J:DO mice (10 male and 10 female, nonsiblings). Since the exome harbors a much smaller percentage of the overall variation in the genome and because founder allele frequencies are balanced in the J:DO population, we estimated that 20 nonsibling J:DO exomes would capture much of the possible protein-coding variation, except for rare/novel variants.

### Genetic variation

We generated exome data from 64 J:ARC mice (32 male and 32 female) representing 32 breeding funnels at G3 and from 20 J:DO mice (10 male and 10 females) at generation 43. There were ∼203 M high-quality reads on average, and after alignment to the reference genome, the mean target coverage was 162 × (91% target covered at 30 × ) for the J:ARC samples and 155 × (90% target covered at 30X) for the J:DO samples. Overall, 90% of the target exome regions are covered by 30 or more reads in both sets of samples (see Supplementary Fig. 2).

Through joint genotyping, we identified 478,011 and 135,825 variants in the J:DO and J:ARC samples, respectively, where a variant is defined as a SNP/INDEL present in a sequenced sample compared with the C57BL/6J, inbred mouse reference genome (mm10, GRCm38). To adjust for the difference in sample size between the J:DO and J:ARC datasets and any potential influence this could have on overall discovery rates, we randomly subset the J:ARC samples to a set of 20 from joint genotyping and then recorded the total number of variants present in the subset. We repeated this analysis over 100 iterations and found from this bootstrapping method that the J:DO harbor, on average, 3.6 × more variants overall than the J:ARC. This higher allelic diversity is expected in the J:DO, because the stock is a multiparent population derived from eight diverse founder inbred strains. Since we posited that a group of 20 nonsibling J:DO mice would harbor most of the available protein-coding variation in the population, we further compared the variant calls generated from low-pass sequencing of 228 DO individuals (Morgan *et al.* 2017) and 69 mouse CC whole genome samples (Srivastava *et al.* 2017). We found 95.2% of DO and 92.6% of the CC protein-coding variants present in our 20-sample call set (Supplementary File 2), highlighting the smaller repertoire of variants found in the coding sequence (vs noncoding sequence), as well as a high-degree of genetic variation present in a relatively small cohort of mice, which is a defining feature of the J:DO population.

### Heterozygosity and inbreeding

**Fig. 2.**
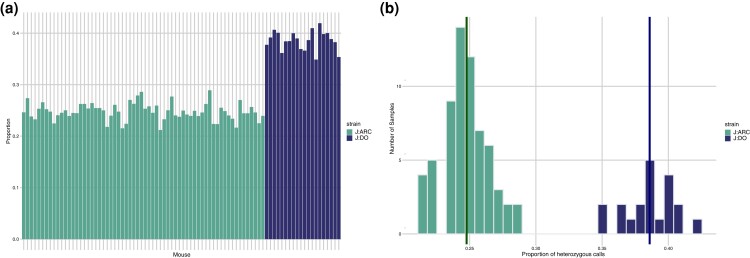
Heterozygosity in the J:ARC (light) and J:DO (dark) datasets. Samples from both datasets are joint called and this analysis is restricted to those shared variants (120,862). The proportion (*a*) of heterozygous calls per sample and distribution of heterozygous calls (*b*) for the J:ARC (light) and J:DO (dark) datasets. The mean heterozygosity for the J:ARC samples is 0.25, while in J:DO, it is 0.39.

To evaluate heterozygosity in these two outbred populations, we estimate sample-level heterozygosity for the entire set of variant calls (Fig. 2a). The average, individual heterozygosity in the J:ARC is 24%, which is consistent with previous estimates from low-pass WGS for CFW outbred populations (Nicod *et al.* 2016; Yalcin *et al.* 2010). In contrast, sample level heterozygosity in the J:DO samples was notably higher (nearly 40%) than the J:ARC samples. Given that previous analyses have estimated J:DO haplotype-level heterozygosity at around 80% (https://www.jax.org/-/media/jaxweb/files/jax-mice-and-services/009376_J_DO_Mar2021.pdf), it is important to note that we used reference-based genotype calls and calculated heterozygosity across the entire call set. Previously, heterozygosity for J:DO mice was calculated on a limited set of variants based on genotype probabilities of eight parental genotypes called from known polymorphic SNPs from the Mouse Universal Genotyping Array (MUGA) series (Morgan *et al.* 2015; Sigmon *et al.* 2020). To recapitulate this haplotype-based approach using our exome variant calls, we used 3,007 calls at markers from the GigaMUGA genotyping array (Morgan *et al.* 2015), and for this subset of variants, we found heterozygosity of ∼82% consistent with the previous array-based estimates (see Supplementary Fig. 3).

**Fig. 3.**
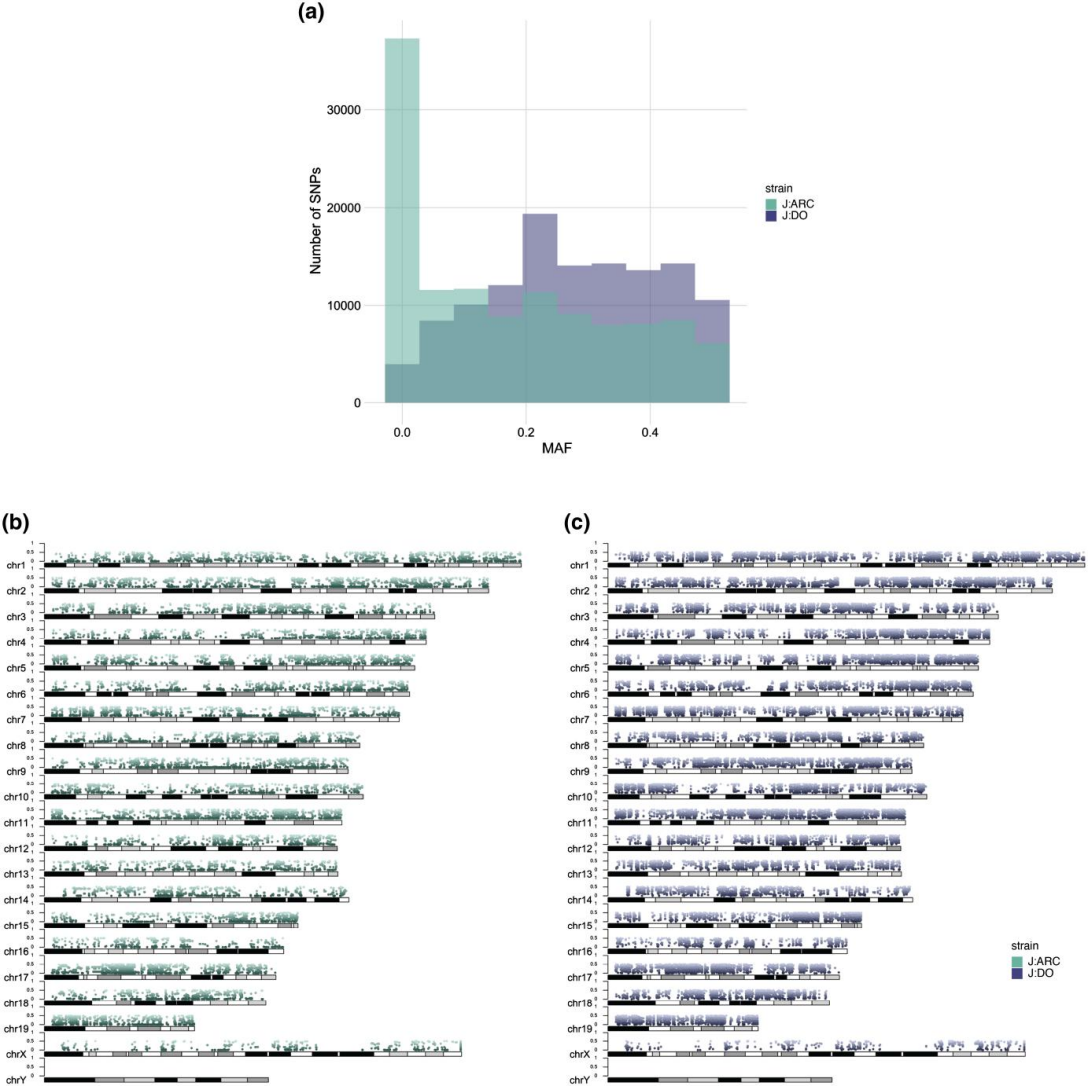
Allele frequency in the J:ARC (light) and J:DO (dark) datasets. Samples from both datasets are joint called and this analysis is restricted to those shared variants (120,862). Allele frequency is calculated using Plink. *a*) MAF distribution for 120,862 SNPs for J:ARC (light) and J:DO (dark). Monomorphic variants are not removed. *b*) Allele frequency at the variant level for the J:ARC samples (mean 0.18, median 0.13). *c*) Allele Frequency for the J:DO samples at the variant level (mean 0.28, median 0.28).

To compare the variant level heterozygosity between the two populations, we identified the variants that are common between J:ARC and J:DO, and then for each variant, we determined the number of samples with heterozygous genotypes (Fig. 2b). We found that a lower proportion of the J:ARC samples are heterozygous for any given variant than the J:DO samples, and that there is greater intraindividual variability in the proportion of heterozygous variants in the J:DO population. To further explore these differences in the distribution of heterozygosity, we calculated the minor allele frequencies (MAF) for each of the common variants across the genome (Figs. 3a–c). We found that for each common variant, the MAF in the J:ARC was consistently lower (mean 0.18, median 0.13) than the J:DO (mean 0.28, median 0.28) and similar to previously published MAF for variants segregating in properly maintained outbred CD-1 mouse populations (Yalcin *et al.* 2010). Since our study design included both male and female individuals, we looked for sex differences in allele frequency for autosomal variants that were found in both populations. To do this, we estimated the XtX statistic, a locus-specific F_ST_ corrected for the covariance of population/grouping allele frequencies. Using this approach, we did not find sex differences in autosomal allele frequency in the J:ARC population. However, in the J:DO, we found some autosomal regions with distinct perturbations of the XtX distribution, though none of these remained significant following BH correction (Supplementary Fig. 4).

We were also interested in the distribution of variants in some regions of the genome, specifically along Chromosome 2 (Chr2), where one parental haplotype was previously shown to cause meiotic drive (known as R2D2) in the J:DO population (Didion *et al.* 2015; Chesler *et al.* 2016; Didion *et al.* 2016). Since this locus was actively removed from the J:DO population, we sought to determine if its removal impacted allelic diversity, locally. To do this, we compared the genetic diversity between Chr2 and the rest of the J:DO exomes in the focal genotypes using a small population genetic analysis. Specifically, we estimated the per-site genetic diversity, or *π*, across the full exome dataset (Danecek *et al.* 2011). The mean of *π* is significantly less for Chr2 ($\bar{\pi }$ = 0.283) vs the whole exome ($\bar{\pi }$ = 0.286; Wilcoxon rank sum test *P* << 0.05), though the overall magnitude of the difference is small. This is likely because of the relatively small size of the missing haplotype (∼15 Mb) compared with all of Chr2 (182 Mb).

**Fig. 4.**
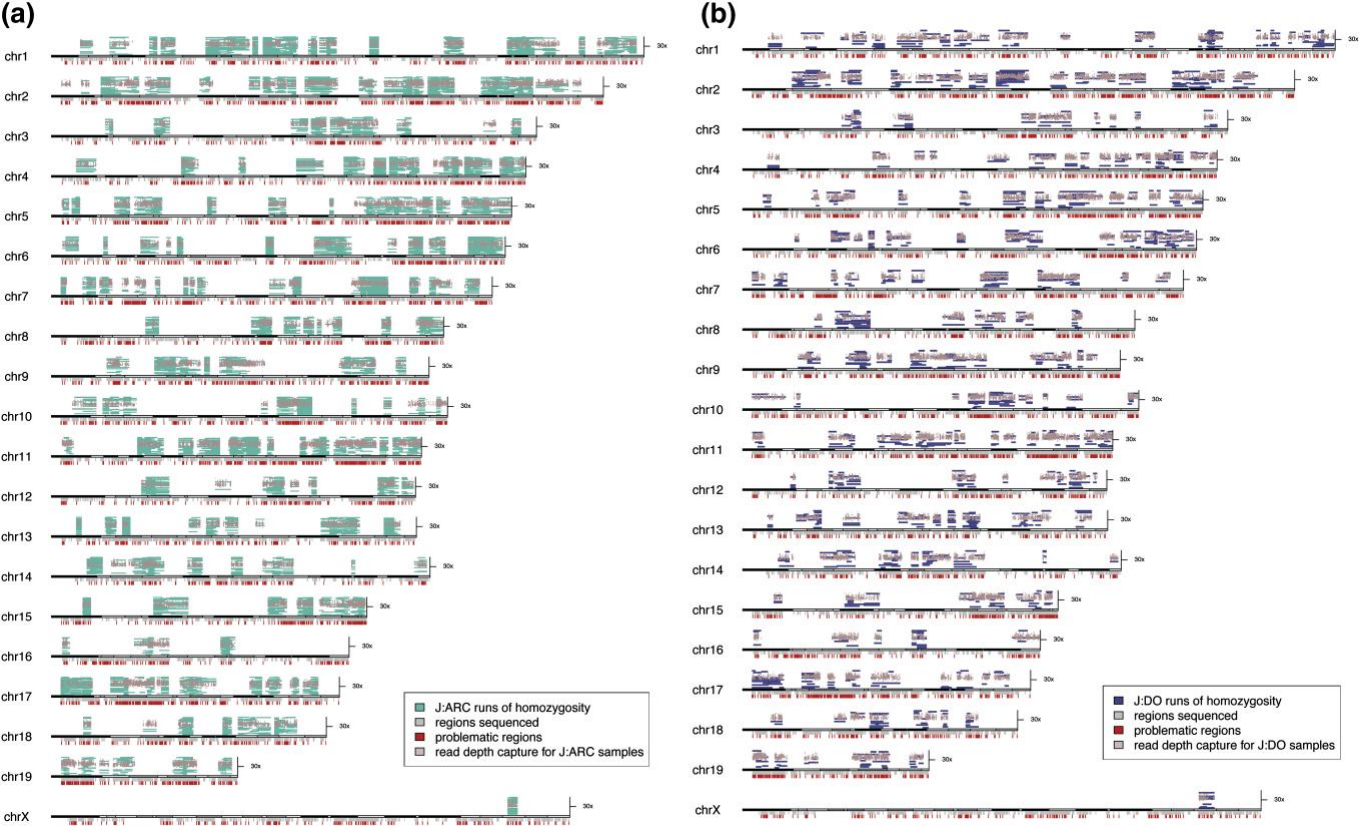
Regions of homozygosity (ROH) for J:ARC (a) and J:DO (b). The number of segments of homozygosity in the J:ARC samples is higher (*a*) than in the J:DO samples (*b*). Plink's default parameters are used. Only runs of homozygosity containing at least 100 SNPs, and of total length ≥1000 kb are noted. Variant calls that are homozygous for the alternate allele in all samples are used for ROH estimation, and the remaining variant calls within the captured regions (light ticks) are used to estimate ROH. Problematic regions of the genome consist of ENCODE-provided regions for mm10 (Amemiya *et al.* 2019), as well as regions annotated to contain segmental duplications, microsatellites, and simple repeats from the UCSC table browser for mm10 (highlighted by dark ticks). The mean coverage per homozygous call position across each ROH is shown (light overlay).

Variant data can also be used to estimate the coefficient of inbreeding (F), which is a metric that describes the distribution of variants in a population. According to the Hardy–Weinberg (HW) principle, F = 0 for a given variant indicates HW equilibrium. By this measure, a fully inbred strain has an inbreeding coefficient of F = 1.0 (100%). When calculated across all the J:ARC variants and samples, the average level of inbreeding is −0.033 (−3.3%), which falls within the expectations for an outbred population (Yalcin *et al.* 2010). Though fewer samples were used to generate the J:DO dataset, the inbreeding coefficient calculated from the J:DO was closer to HW equilibrium at −0.007 (−0.7%). These F coefficients indicate that both populations are maximally outbred. The negative values are the result of heterozygous calls that exceed HW expectations, which in these datasets could be due to erroneous genotype calls or under sampling of the population. While the coefficients of inbreeding for these populations are low, we found regions that contain stretches of homozygosity (ROH). These regions of homozygosity are distributed through the J:ARC and J:DO genomes (Fig. 4). To a certain extent, these ROH overestimate true homozygosity. For example, some of these regions could be hemizygous due to deletions. We looked for significant drops in coverage across these regions, but found that coverage across these regions falls within our mean coverage range for each population (Fig. 4). However, exome sequencing does not reliably distinguish potential hemizygous and true homozygous variant calls; therefore, this is one source of ROH overestimation that will require further analysis using sequencing technologies that are better suited for SV detection (e.g. long-read sequencing). Another known source of ROH overestimation is reference bias where private variation or poorly assembled regions in the reference genome lead to pervasive false positive homozygous variant calls in unrelated samples (variants with no MAF). As shown in Fig. 4, many of the ROH do overlap with problematic regions of the reference genome.

### Interindividual variability

**Fig. 5.**
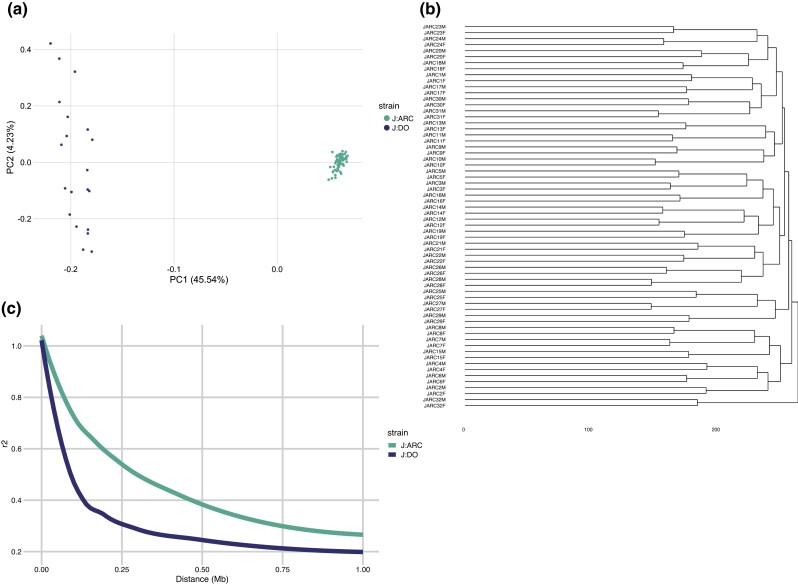
Interindividual variability, relatedness, and LD decay. *a*) The PCA plot of J:DO (dark) and J:ARC (light) joint variant calls. *b*) A dendrogram of the J:ARC samples (using all called variants in the dataset) showing samples clustering in known sibling relationships. *c*) Comparison of LD decay J:ARC samples (light) and J:DO samples (dark). Each curve is plotted using the 95th percentile of r2 values for SNPs spaced up to 1 Mb apart. Plink is used to calculate *r^2^* for all pairs of autosomal SNPs called from joint genotyping in the J:ARC samples (425,409) and the J:DO samples (117,429). SNPs that are missing in more than 5% of the samples and are monomorphic are removed.

An important consideration in selecting an outbred laboratory mouse strain and sample size in experimental design is interindividual genetic variability. Taking advantage of our variant data, we used PCA to assess interindividual and interstrain variation in the J:ARC and J:DO. Using all jointly called variants, we found the expected interstrain variation (PC1) but more interindividual variability in the J:DO samples than the J:ARC samples (PC2) (Fig. 5a). These data show that greater genetic diversity can be achieved with fewer samples in the J:DO by at least an order of magnitude. Unbiased clustering of the J:ARC variant data recapitulated the known sibling relationships of the J:ARC samples (Fig. 5b), highlighting the utility of variant data for pedigree reconstruction in outbred populations.

### Linkage disequilibrium decay

Linkage disequilibrium (LD) describes the degree to which variants co-segregate in a population. Meiotic recombination leads to new haplotypes and is the molecular mechanism that drives LD decay. Pairwise analysis of linked markers in an interval reveals LD decay in a population and these data are useful for inferring swept radius for marker spacing in genetic association studies. Higher LD decay in a mapping population provides higher resolution for genetic mapping. We examined this facet of genetic architecture in the J:ARC and J:DO populations, and while our sample size is low, we found that both populations offer LD decay sufficient for genetic mapping, with higher LD decay in the J:DO (LD_1/2_ = ∼0.1 Mb) (Fig. 5c). Our estimate of LD decay is higher than the previous estimates for the J:DO, but this is consistent with the more advanced breeding generation used in our study (Gatti *et al.* 2014). LD decay in the J:ARC is consistent with the previous estimates for commonly used laboratory outbred populations (LD_1/2_ = ∼0.3 Mb) (Yalcin *et al.* 2010).

### Novel variants

**Fig. 6.**
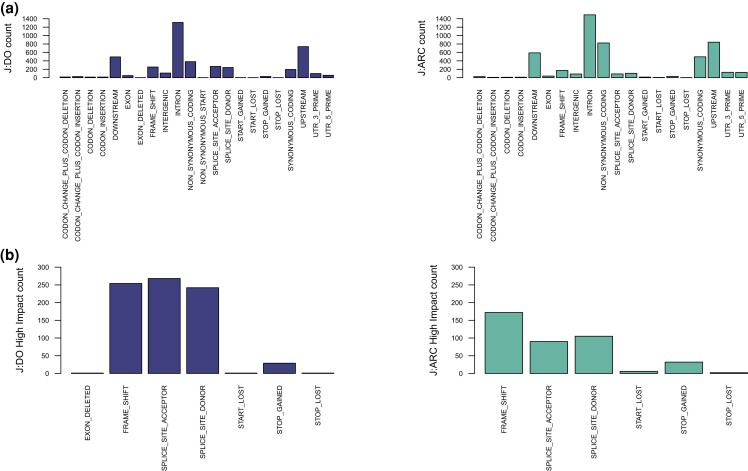
Novel variant consequences. *a*) The SnpEff effect categories for novel variants for J:DO (left) and J:ARC (right) (*b*) High-impact (SnpEff effect categories) novel variants are enriched in J:DO (left) in comparison to J:ARC (right).

To identify the novel variant calls in the J:ARC and J:DO, we compared the calls to all published variants from all sequenced laboratory mouse strains in dbSNP, EVA, the Sanger mouse genomes project (Lilue *et al.* 2018), Mouse CC Genome (Srivastava *et al.* 2017), and to in-house J:DO genome variant calls from low-pass sequencing of 228 individuals (Morgan *et al.* 2017). Of the 135,825 and 478,011 variants found in the J:ARC and the J:DO, respectively, 96.24 and 99.08% are variants that have been identified in other laboratory mouse strains. We identified 5,105 [2,191 > AF 0.2] and 4,308 [1,888 > AF 0.2] novel variants, respectively (Table 1). The larger number of novel variants in J:ARC mice is attributable to the relative lack of published variant data from the Swiss-derived outbred stocks compared with the complete catalog of variation available from the eight founder inbred strains of the CC recombinant inbred panel from which the J:DO were derived. Functional annotation of the novel variants reveals that the J:DO have nearly twice the number of protein-damaging variants (including exon deletions, frameshift, stop gain/loss, splice acceptor/donor sites, start loss) [J:DO:797 (329 > AF 0.2, HIGH), J:ARC: 408 (151 > AF 0.2, HIGH)] (Fig. 6). The details of each variant are provided in Supplementary File 3. The genes harboring these high-impact variants in the J:DO show functional enrichment for post-translational modifications (Ubl conjugation, isopepeptide bond), DNA repair, and translational regulation/RNA binding; while the genes harboring high-impact variants in the J:ARC do not show functional enrichment. The novel protein damaging variants in the J:DO are variants that have naturally arisen in the population, but have not been lost through purifying or artificial selection. These genetic differences are attributable not only to the distinct origins and ages of these outbred stocks, but also to the different breeding paradigms and selective pressures that have occurred during their maintenance. For example, many outbred mouse stocks and populations have been subjected to artificial selection for desirable laboratory traits (fecundity, docility, size, etc.) leading to a lack of variability for some phenotypes. One example is body weight where variation is less desirable because it complicates pharmacological applications, i.e. dosing studies. As a result, body weight variation in CD-1 and related outbred like the J:ARC is minimized; however, this is not the case in the J:DO, where a larger range of body weights is observed (Supplementary Fig. 5).

**Table 1.** Total variants (SNPs/INDELs) and total novel variants in J:DO and J:ARC stocks.

Stock	Type	Total variants	Mouse collaborative cross	Diversity outbred mouse genomes	SangerSNP_Indel _May_2020	dbSNP	EVA	Novel	High impact novel variants
**J:DO**	SNP	425,409	421,107	387,944	418,032	398,929	398,862	1,563	67
	INDEL	50,511	45,464	38,596	33,332	39,011	39,039	2,668	718
	MIXED	2,091	1,916	1,389	1,625	1,742	1,741	77	12
**J:ARC**	SNP	117,429	104,794	99,398	111,524	102,145	102,124	3,331	79
	INDEL	17,783	11,703	10,238	9,551	13,046	13,071	1,676	319
	MIXED	613	352	172	320	366	366	98	10

A comparison of the total variants to all variants in the major, publicly available mouse variant resource reveals that 0.9 and 3.75% of the total variants found in the J:DO and J:ARC, respectively, are novel.

## Conclusions

We used exome sequencing to define protein-coding variation in two outbred populations of mice with distinct origins. We found that the multiparent, J:DO outbred population has more than 3 × population-level variation than the CD-1 derived J:ARC outbred population. Both populations have coefficients of inbreeding that are consistent with the previous estimates, confirming that both are maximally outbred. Our analysis of variants that are shared between the J:ARC and J:DO shows that individual heterozygosity in both populations is high, but allelic diversity, intraindividual variability, and MAF are higher in the J:DO population. For research applications, this means that similarly sized cohorts of J:DO mice will provide more genetic diversity than J:ARC mice, making the former population a better choice for studies that endeavor to maximize genetic diversity. Our data and multiple previous studies show that both outbred populations are useful for genetic mapping. But a higher genome-wide LD decay in the J:DO will confer higher mapping resolution.

Overall, we found more novel variants in J:ARC compared with J:DO, and this is likely due to the relative paucity of published/accessible variant data for commonly used outbred strains, especially CD-1 from which the J:ARC population was derived. While there were fewer novel variants in the J:DO population, more of these were variants that are predicted to be protein damaging and potentially more likely to reduce survival and/or fecundity. While this study does not allow us to make any inferences about load of deleterious alleles and their respective contributions to fecundity, litter size is a trait that is frequently used to estimate fecundity. Breeding data from The Jackson Laboratory show that J:DO females have litter sizes that are comparable to many common laboratory inbred strains (mean litter size = 9.9, Supplementary Fig. 5). The J:ARC population is derived from an outbred stock that has been subject to nearly a century of artificial selection for high fecundity (mean litter size = 15.4, Supplementary Fig. 5), docility, and other desirable laboratory traits like low intrastrain body weight variability which is preferred for dosing studies. In contrast, the J:DO population is derived from phenotypically diverse recombinant inbred lines (CC) and artificial selection for any phenotype is actively avoided through blinded, random assignment of breeders. These differences in selective pressure may also explain the apparent loss of deleterious alleles through drift in the J:ARC. Deleterious alleles that have not been subject to purifying selection in the J:DO, also highlight the potential buffering effects of high allelic diversity and heterozygosity.

Our data, together with the previously published studies, reinforce our recognition that not all outbred stocks have comparable genetic and phenotypic diversities. Direct sequencing of outbred stocks provides a molecular basis for genetic quality control, breeding paradigms, and optimization of experimental design for the effective deployment of these strains in biomedical research.

### Study limitations

Our exome sequencing data provide just a subset of the overall genetic variation segregating in these outbred populations, specifically SNPs and small insertions/deletions in the coding sequence. Structural variation, which we have not profiled here, is also a significant source of genetic variation. SV has the potential to impact multiple genes with potentially large phenotypic effects. While certain types of structural variants (deletions) can be short-read whole genome sequencing data, long-read sequencing, and de novo assembly are the gold standards for the genome-wide detection of all types of SV. Moreover, outside of splice junction sites and UTRs, our data do not capture non-coding variation, yet non-coding variation is also a recognized source of phenotypic variation, especially for complex traits. Finally, while our sequencing depth per sample is high, our sample sizes are low, making our study underpowered for the detection of rare SNPs/INDELs.

## Supplementary Material

jkad015_Supplementary_Data

## Data Availability

All the sequence data used in this study have been submitted to NCBI Sequence Read Archive under the bioproject PRJNA835415 and study SUB11284953. Supplemental material is available at G3 online.
