# Supplementary material for: Protein coding variation in the J:ARC and J:DO outbred laboratory mouse stocks provides a molecular basis for distinct research applications
Source: G3 (Bethesda). 2023 Jan 17;13(4):jkad015. doi: 10.1093/g3journal/jkad015 (PMC10085793; doi:10.1093/g3journal/jkad015)
Supplement: jkad015_Supplementary_Data [file jkad015_supplementary_data.zip › Supplementary_Material_Legends_G3-2023-404045.docx]

### SUPPLEMENTARY MATERIAL

**Supplementary File S1.** J:DO and J:ARC samples used for the study.

**Supplementary File S2.** Comparison of exome variant calls in the J:DO samples to variant calls from previously published DO and Collaborative Cross sequencing projects (Morgan *et al.* 2017; Srivastava *et al.* 2017). Ninety-five (95%) of exomic variants found in the 228-sample call set were also found in the 20 J:DO samples. Since the 228 DO call set was from low pass sequencing, the J:DO variant calls were also compared to exomic variant calls from 69 Collaborative Cross lines (from which the DO population were derived) and 92.6% overlap was found.

**Supplementary File S3.** All novel, protein-coding variants in J:ARC and J:DO with functional annotation and allele frequency. Functional annotation (DAVID Bioinformatics Resources) clustering of genes harboring novel variants in J:ARC and J:DO.

**Supplementary Figure S1.** The Jackson Laboratory (JAX) Nextflow pipeline for laboratory mouse whole exome sequencing.

**Supplementary Figure S2.** Exome sequencing coverage statistics. **(A)** The mean target coverage versus high quality filtered reads showing mean target coverage was 162X (91% target covered at 30X) for J:ARC samples (green) and 155X (90% target covered at 30X) for J:DO (blue) samples. **(B)** Percent target bases at 30X versus high quality filtered reads showing 90% of the target exome regions were covered by 30 or more reads in both sets of samples.

**Supplementary Figure S3.** The proportion of heterozygous variants based on the subset of variant calls for known SNPs within the Mouse Universal Genotyping Array probe set. There were 3007 exome variant calls at positions that are included in the GigaMUGA probe set. These were used to estimate the proportion of heterozygous calls per J:DO using the R package, R/qtl2.

Estimates are based on autosomes at the haplotype level, using genotype probabilities of the 8 J:DO parental inbred strain genotypes.

**Supplementary Figure S4.** Comparison of sex differences in allele frequency for autosomal variants. The XtX statistic, a locus-specific F_ST_ corrected for covariance of population/grouping allele frequencies was calculated for all biallelic variants called in both populations. A histogram of p-values for the XtX summary was assessed prior to false discovery rate (FDR) control. **(A)** For the J:ARC population, there were no differences in allele frequency between males and females as shown by the distribution of XtX and the resultant p-values. **(B)** For J:DO, there were genomic regions that deviated from the uniform distribution of XtX, though none of these variants remained significant following BH correction.

**Supplementary Figure S5.** Distribution of body weight and mean litter size in J:ARC and J:DO stocks. **(A)** Jackson Laboratory data (unpublished, Schile 34608) and publicly available datasets from the Mouse Phenome Database ((Shorter8, Chesler4 (Logan *et al.* 2013; Recla *et al.* 2014), <https://phenome.jax.org/>) and Wright et al. (Wright *et al.* 2022) were used to assess the distribution of body weight in J:DO and J:ARC mice). Data include both males and females and sub-sets of 60 were randomly sampled from larger datasets for comparison. Overall body weight in the J:ARC is higher than J:DO (ANOVA, adjusted P value <0.0001), while the distribution of body weight (range) is greater in J:DO. **(B)** Litter size data from 1,620 litters were recorded from 01/01/2022 to 07/19/2022. Data included 0 - 2 litters for all dams in the breeding colonies at The Jackson Laboratory (i.e. unproductive dams included). The cumulative distributions were non-normal and were compared using a non-parametric t test (Kolmogorov-Smirnov), approximate P value was <0.0001.
