# Supplementary figures and images for: Protein coding variation in the J:ARC and J:DO outbred laboratory mouse stocks provides a molecular basis for distinct research applications
Source: G3 (Bethesda). 2023 Jan 17;13(4):jkad015. doi: 10.1093/g3journal/jkad015 (PMC10085793; doi:10.1093/g3journal/jkad015)

SUPPLEMENTARY FIGURE S1

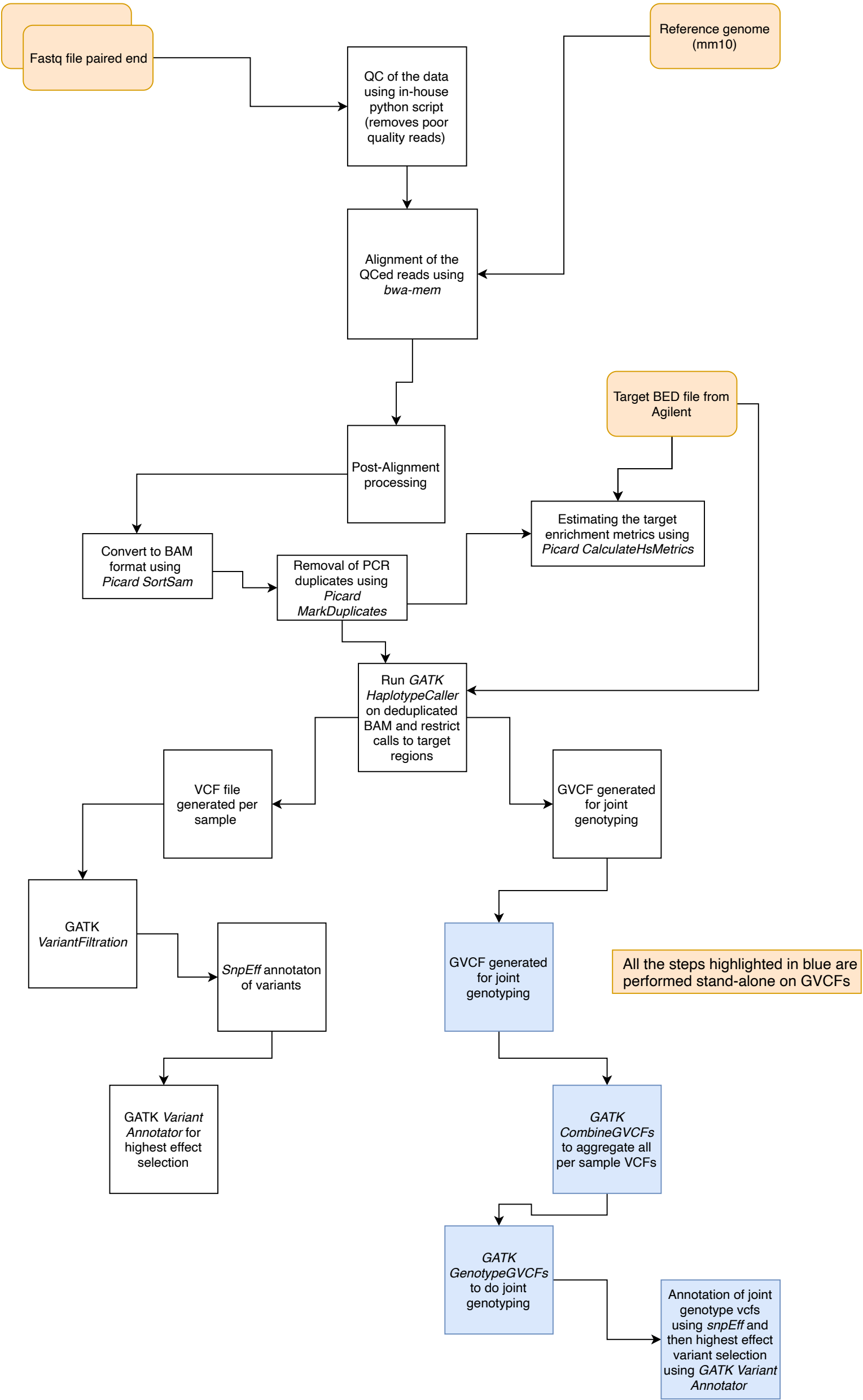

Supplement: jkad015_Supplementary_Data [file jkad015_supplementary_data.zip › Supplementary_Figure_S1_G3-2023-404045.pdf]

SUPPLEMENTARY FIGURE 2

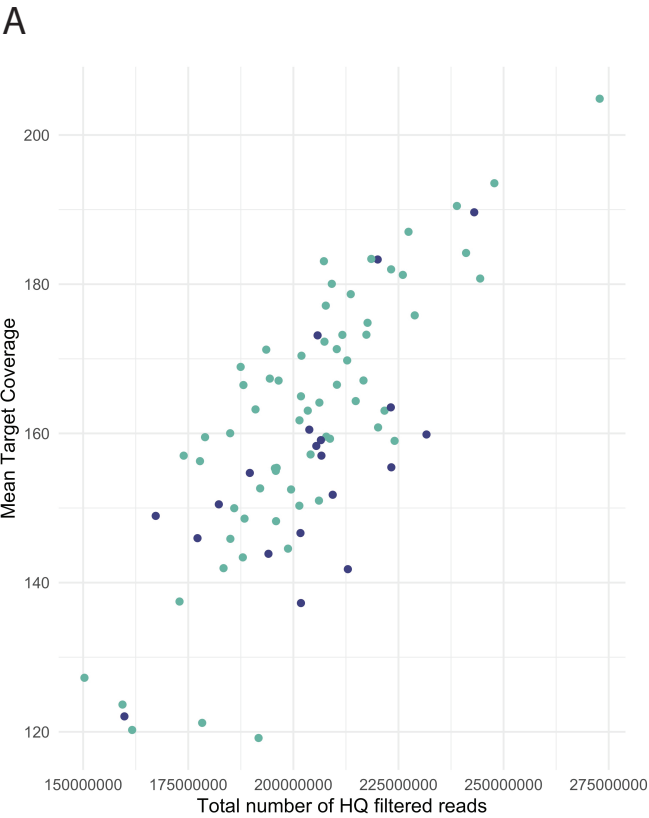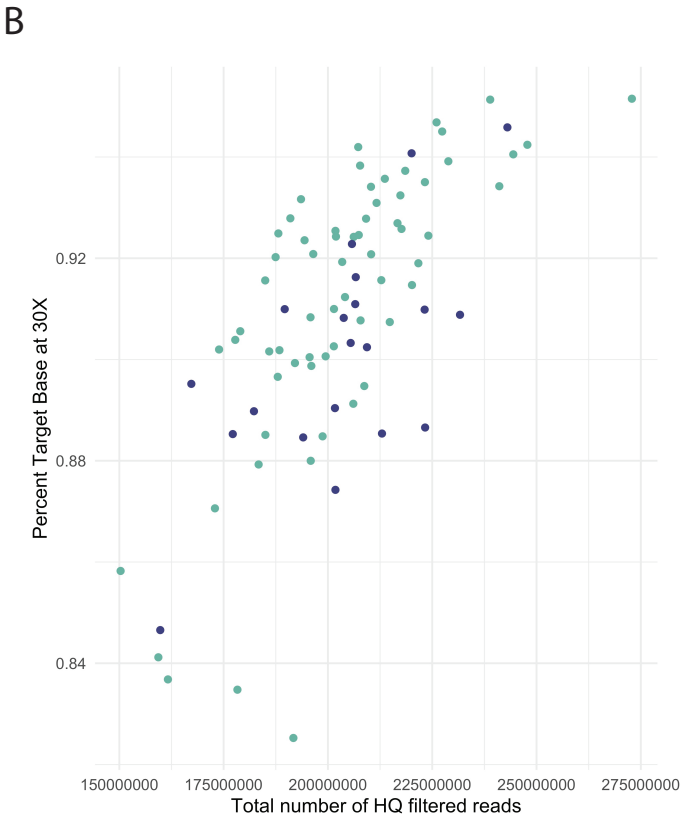

Supplement: jkad015_Supplementary_Data [file jkad015_supplementary_data.zip › Supplementary_Figure_S2_G3-2023-404045.pdf]

SUPPLEMENTARY FIGURE S3

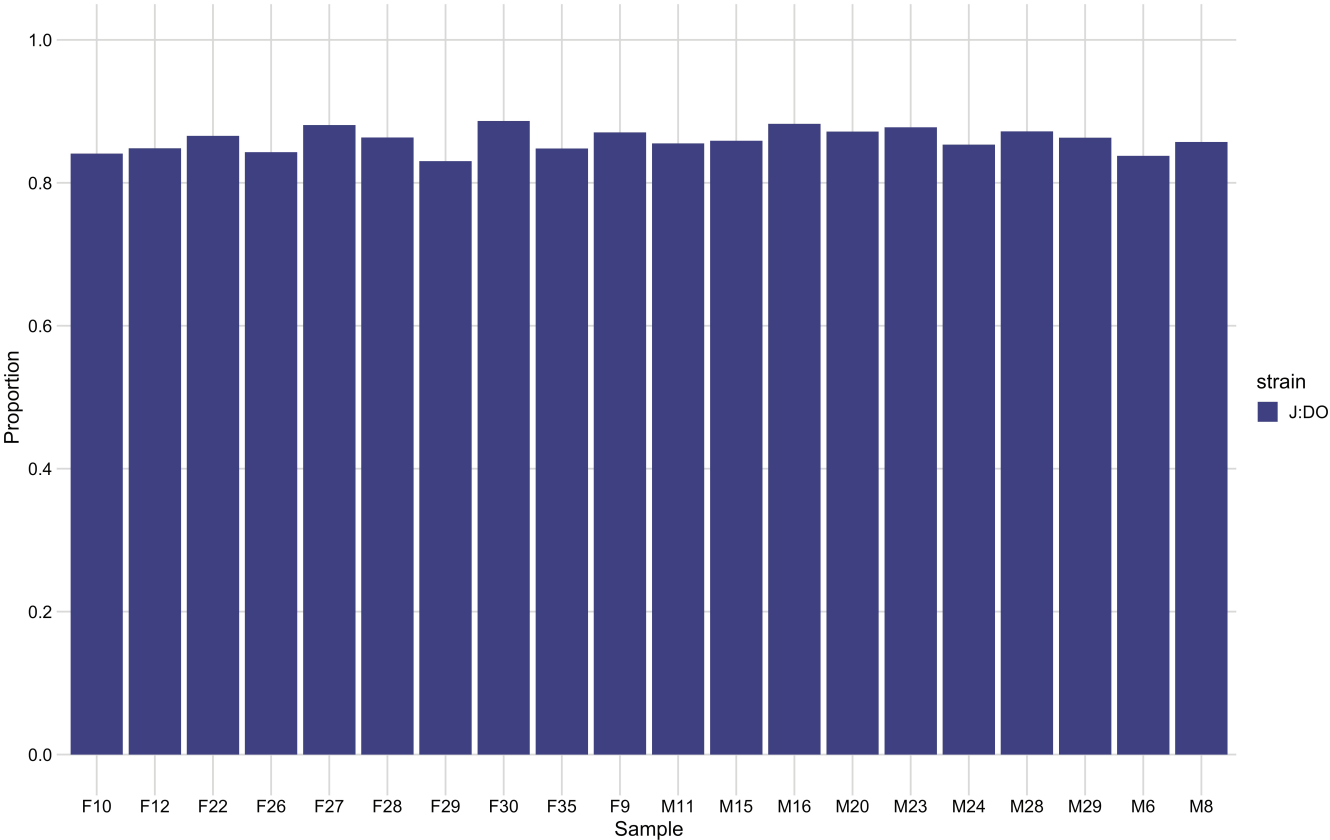

Supplement: jkad015_Supplementary_Data [file jkad015_supplementary_data.zip › Supplementary_Figure_S3_G3-2023-404045.pdf]

A

J:ARC

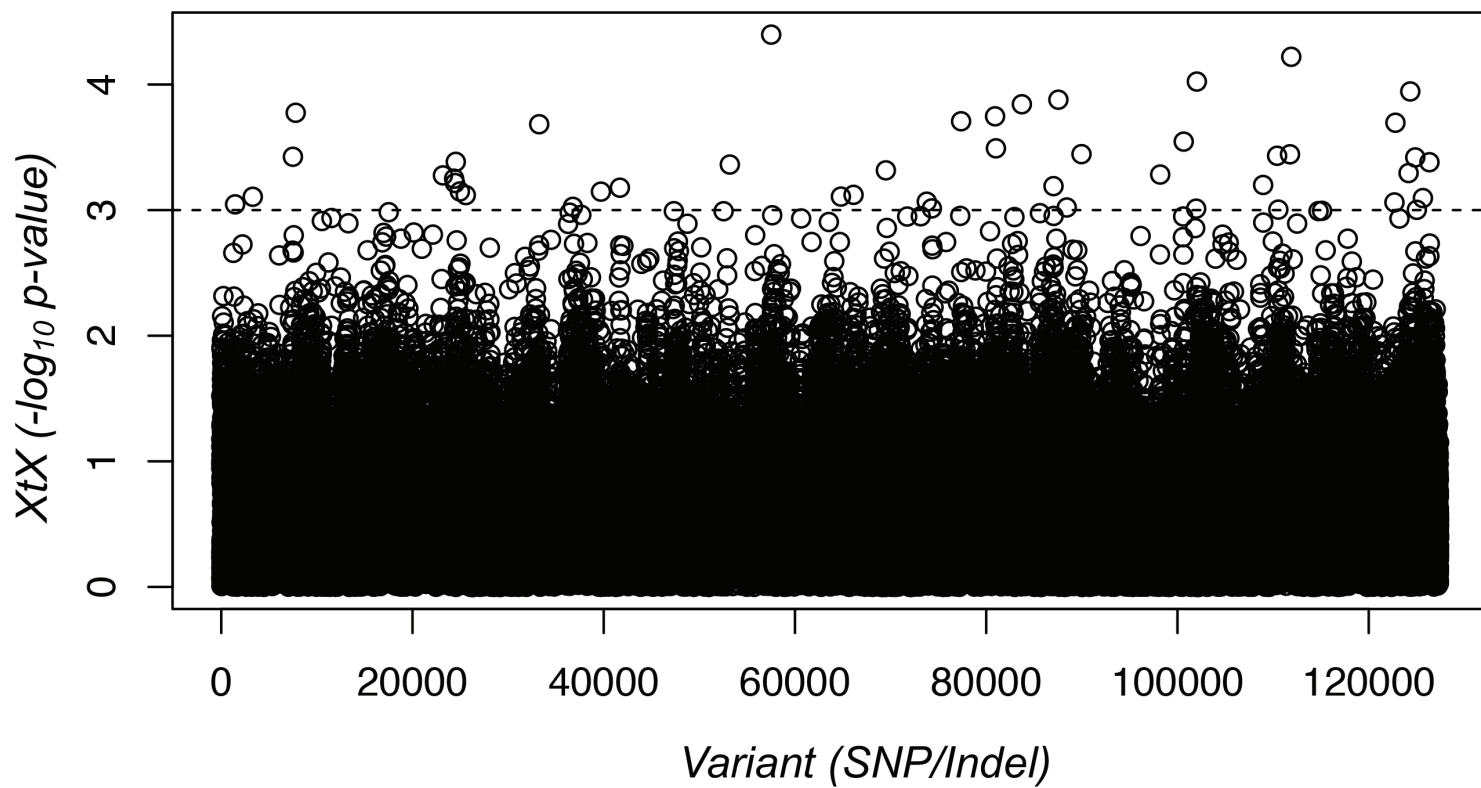

B

J:DO

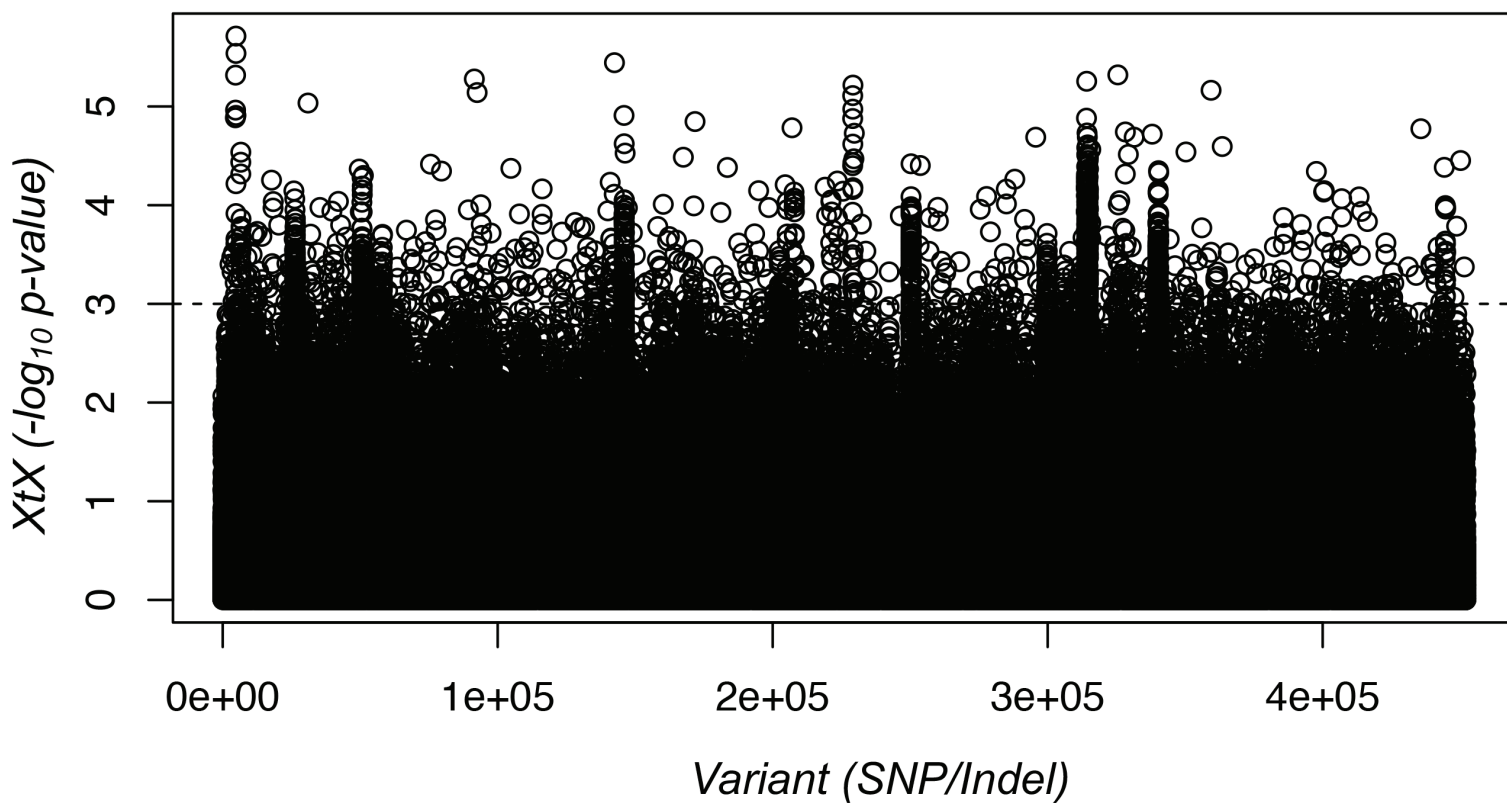

Supplement: jkad015_Supplementary_Data [file jkad015_supplementary_data.zip › Supplementary_Figure_S4_G3-2023-404045.pdf]

SUPPLEMENTARY FIGURE S5

A

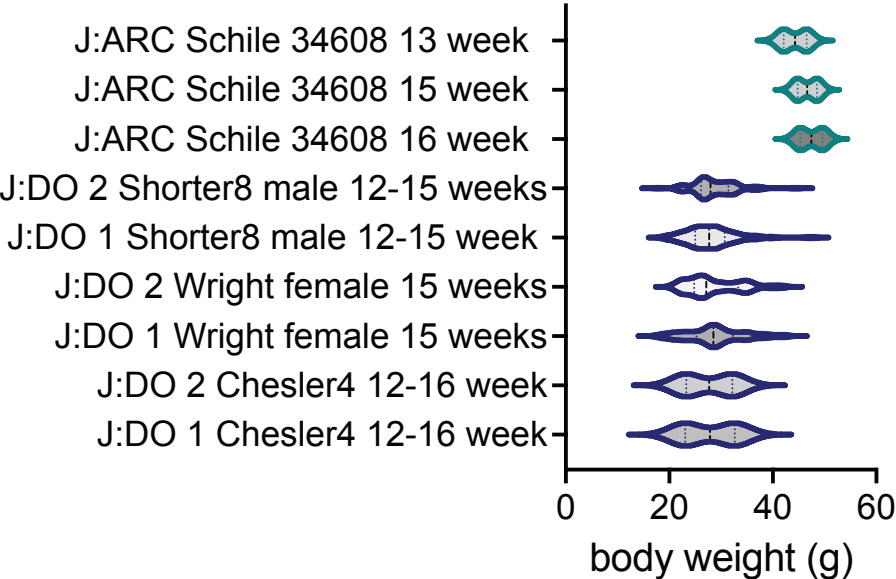

B

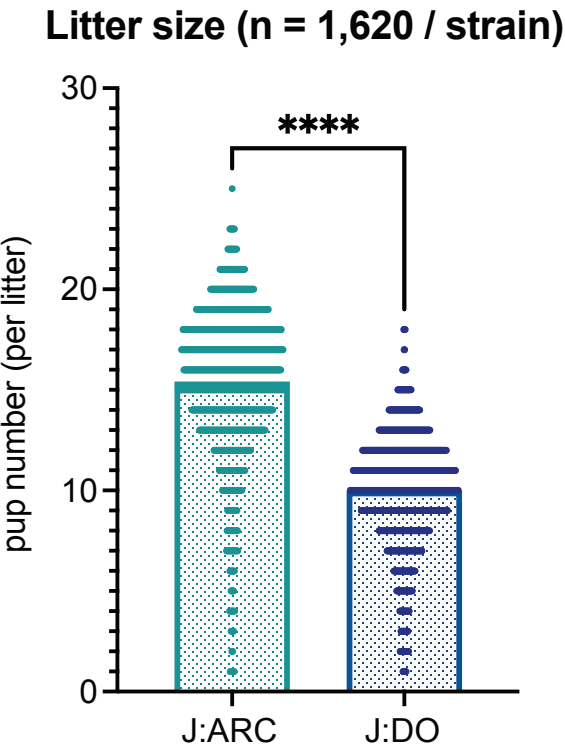

Supplement: jkad015_Supplementary_Data [file jkad015_supplementary_data.zip › Supplementary_Figure_S5_G3-2023-404045.pdf]
